# Detection of pagetoid urothelial intraepithelial neoplasia extending to the vagina by cervical screening cytology: a case report with renewed immunochemical summary

**DOI:** 10.1186/s13000-019-0788-2

**Published:** 2019-02-02

**Authors:** Yuki Koyanagi, Chiaki Kubo, Shigenori Nagata, Ayumi Ryu, Koji Hatano, Rieko Kano, Satoshi Tanada, Jun-ichi Ashimura, Atsushi Idota, Shoji Kamiura, Tomoyuki Yamasaki, Shin-ichi Nakatsuka

**Affiliations:** 1 Department of Clinical Laboratory, Osaka International Cancer Institute Hospital, Osaka, Japan; 2 Department of Diagnostic Pathology and Cytology, Osaka International Cancer Institute Hospital, 3-1-69 Otemae, Chuo-ku, Osaka, 541-8567 Japan; 3 Department of Urology, Osaka International Cancer Institute Hospital, Osaka, Japan; 4 Department of Gynecologic Oncology, Osaka International Cancer Institute Hospital, Osaka, Japan

**Keywords:** Pagetoid urothelial intraepithelial neoplasia, Secondary paget disease, Urothelial carcinoma, Bladder cancer, Lower genital tract, Vagina, GATA3, p16/Ki-67 double labeling, Cervical screening cytology, Liquid-based cytology

## Abstract

**Background:**

Pagetoid spread of urothelial carcinoma (UC) to the lower genital tract is quite a rare and diagnostically challenging condition. Pagetoid urothelial intraepithelial neoplasia extending to the vagina is difficult to diagnose, especially in remote recurrences without symptomatic or macroscopic lesions typical to Paget disease. However, its identification by cervical screening cytology is important because UC is often characterized by a long history of relapse.

**Case presentation:**

A 68-year-old Japanese postmenopausal woman developed brown vaginal discharge after radical cystectomy for bladder cancer (high-grade UC, pT2a pN0 cM0 [Union for International Cancer Control, 8th edition]) concomitant with focal in-situ UC in the urethra. She had a history of left renal pelvis UC, which was surgically removed 9 months before the radical cystectomy. Gynecologic examination of the lower genital tract was unremarkable although cervical screening cytology demonstrated severely atypical cells with pleomorphism repeatedly. Cervical colposcopy and diagnostic conization revealed no cervical neoplasm. In retrospect, immunocytochemical p16/Ki-67 dual staining for the previous cervical screening was negative for p16 labeling, and the neoplastic cells were positive for cytokeratins 7 and 20, p63, and GATA binding protein 3. No high-risk human papillomavirus genotype was identified by an automated DNA chip system using liquid-based cytology samples. Eleven months post-cystectomy, punch biopsy of the vulva and vagina confirmed intraepithelial UC in the juxtaposed squamous epithelium with pagetoid spread demonstrating positivity for specific urothelial markers: uroplakins II and III and thrombomodulin. Concurrent invasive malignancy was ruled out, and CO_2_ laser vaporization of the vulvar and vaginal lesion was performed. The patient remained alive without evidence of invasive malignancy for 14 months after the radical cystectomy for bladder cancer.

**Conclusions:**

To detect recurrent pagetoid urothelial intraepithelial neoplasia with pagetoid spread in the lower genital tract, pathologists should recognize the history of prior UC with special attention to absence of p16 labeling in cervical cytology as a pointer to the diagnosis of urothelial cancer. Using further biopsy and immunohistochemical confirmation of UC relapse, investigation to rule out invasive malignancies and careful follow-up throughout the patient’s lifetime is recommended.

## Background

Extensive urothelial carcinoma (UC) involves various organs along the urinary tract and often has a long history of relapse [1]. Pagetoid spread of UC to the lower genital tract is rare and difficult to diagnose, especially in remote recurrences without apparent macroscopic lesions. We describe a case of metachronous UC with pagetoid spread to the vagina, which was diagnosed by cervical cytology and vaginal biopsy, and herein summarize the clinicopathologic differences among Paget-like neoplasms of the lower genital tract with recent immunocytochemical/immunohistochemical markers.

## Case presentation

### Clinical course

**Fig. 1:**
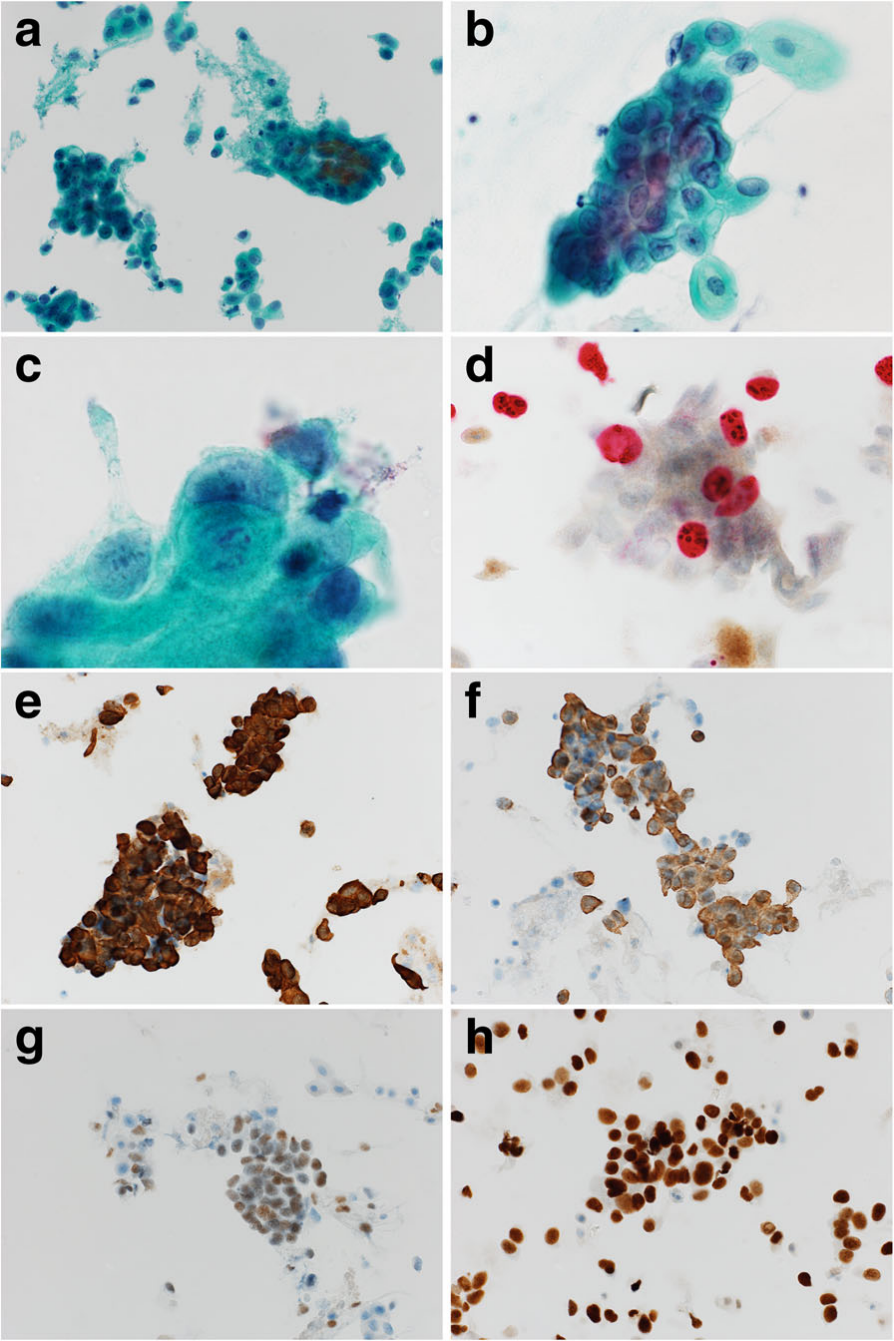
Photomicrographs of pagetoid urothelial intraepithelial neoplasia extending to the vagina. **a**–**h** Liquid-based cytology by vaginal scraping. **a** Stratified or linear clusters of atypical cells with partially necrotic changes (Papanicolaou [Pap] stain, × 200). **b** Cluster exhibiting nuclear stratification and disarrangement with cytoplasmic vacuoles and enlarged nucleoli (Pap, × 400). **c** Cell cannibalism in the margin of the cluster (Pap, × 1000). **d** CINtec® *PLUS* test demonstrating positive red nuclear labeling with Ki-67, but weak brown staining for p16 considered negative (immunocytochemical [ICC] stain, × 400). **e** Positivity for CK7 (ICC, × 200). **f** Positivity for CK20 (ICC, × 200). **g** Positivity for p63 (ICC, × 200). **h** Positivity for GATA3 (ICC, × 200)

**Fig. 2:**
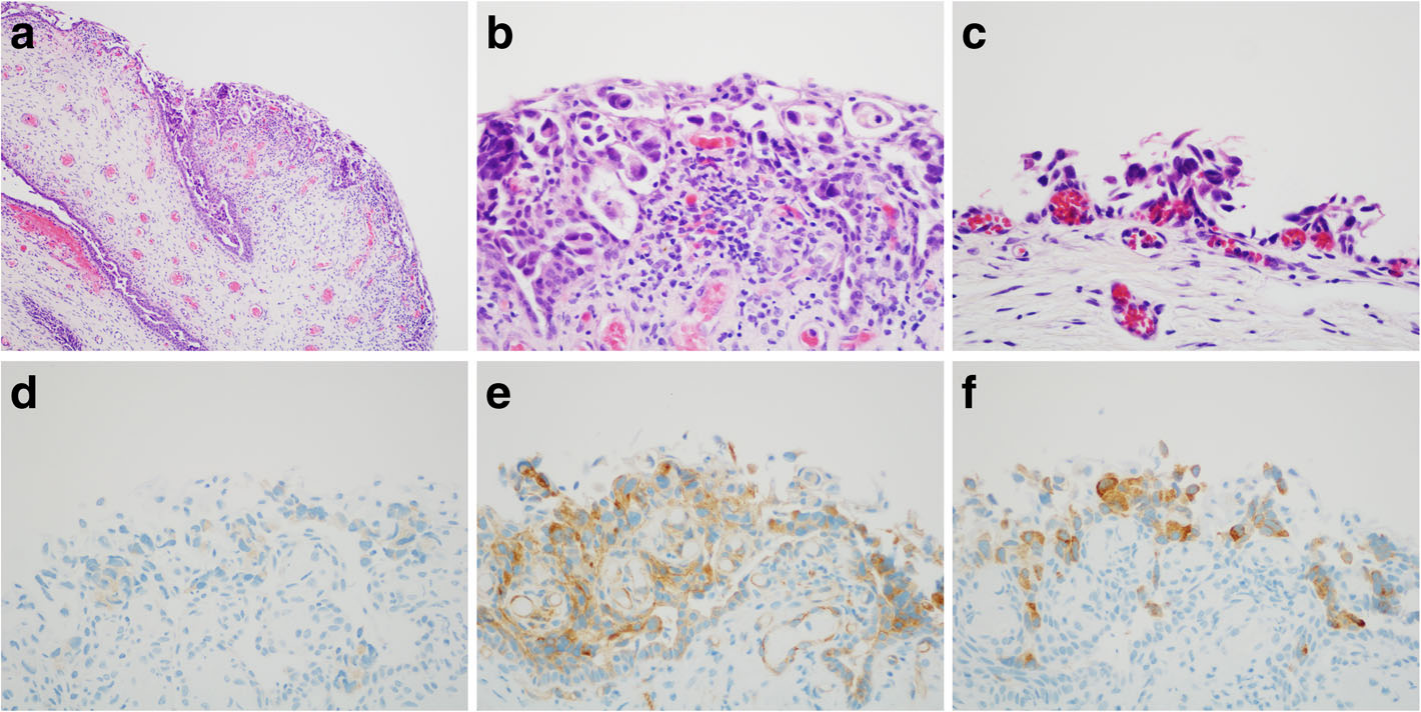
Photomicrographs of pagetoid urothelial intraepithelial neoplasia extending to the vagina. **a**–**f** Punch biopsies from the vulva and anterior vaginal wall. **a** Large cells proliferating within the basal and parabasal layers of the vulvar squamous epithelium (hematoxylin and eosin [HE] stain, × 10). **b** Paget-like cells with larger nuclei and paler cytoplasm than the adjacent keratinocytes about clefts, an apparent artifact that is a common finding in pagetoid urothelial intraepithelial neoplasia (HE, × 400). **c** Similar cells of high nuclear grade identified in the mostly denuded vagina (HE, × 400). **d** Focal positivity for uroplakin III (immunohistochemical [IHC] stain, × 400). **e** Positivity for thrombomodulin (IHC, × 400). **f** Positivity for uroplakin II (IHC, × 400)

A 68-year-old Japanese postmenopausal woman developed brown vaginal discharge after radical cystectomy for bladder cancer, which was histologically diagnosed as high-grade UC invading up to the superficial muscularis propria without metastasis (pT2a pN0 cM0 [Union for International Cancer Control, 8th edition]) concomitant with focal urothelial carcinoma in situ in the urethra. She had a history of left renal pelvis UC, which was surgically removed after repeated 80-mg/fraction Bacillus Calmette-Guérin injections via a single J stent, resulting in no residual carcinoma 9 months before the radical cystectomy. She was also treated for breast cancer at 47 years of age without postoperative recurrence. Gynecologic examination of the lower genital tract, including the periurethral region, was unremarkable; however, cervical screening cytology demonstrated severely atypical cells suspicious for adenocarcinoma (Fig. 1a-c). Cervical colposcopy and diagnostic conization revealed no cervical neoplasm. Eleven months post-cystectomy, punch biopsy of the vulva and vagina confirmed intraepithelial UC in the juxtaposed squamous epithelium with pagetoid spread (Fig. 2a-c). Concurrent invasive malignancy was ruled out, and CO_2_ laser vaporization of the vulvar and vaginal lesion was performed. The patient remained alive without evidence of invasive malignancy for 14 months after the radical cystectomy for bladder cancer.

### Immunocytochemical and immunohistochemical analyses and human papillomavirus test

The CINtec® *PLUS* cytology test (Roche Diagnostics, Basel, Switzerland), an immunocytochemical p16/Ki-67 dual staining kit for screening of cervical disease, was negative for p16 labeling (Fig. 1d). Immunocytochemistry revealed neoplastic cells positive for cytokeratin (CK) 7, CK20, p63, and GATA3 (Fig. 1e-h). Immunohistochemical examination of the biopsy sample of the vulva and vagina revealed neoplastic cells positive for uroplakin III, thrombomodulin, and uroplakin II (Fig. 2d-f) but negative for carcinoembryonic antigen (CEA), gross cystic disease fluid protein 15 (GCDFP15), and S100. No high-risk human papillomavirus (HPV) genotype was identified by an automated DNA chip system (Clinichip™ HPV test; Sekisui Medical, Tokyo, Japan) using LBC samples.

## Discussion

Female genital organs can be synchronously or metachronously involved in bladder cancer, with vaginal involvement predominating. Recently, Salem et al. reported on 360 women who underwent radical cystectomy for bladder cancer, of which 13 (3.6%) had vaginal involvement and 1 had uterine spread [2]. Similarly, Djaladat et al. found vaginal involvement in at least 10 (3.7%) of 267 women who underwent cystectomy with reproductive organ removal [3]. Although the morphologic pattern of vaginal involvement is unclear in those studies, pagetoid spread of UC is considered much rarer; Gregori et al. reported that only 2 of 98 cases of vulvar Paget disease were related to bladder UC [4]. In the present case, biopsy revealed both the vulvar and vaginal involvement by UC whereas there was no evidence of uterine involvement in the specimen of cervical conization.

Pagetoid spread of carcinoma is characterized by proliferation of individual or clustered tumor cells in the lower part of the juxtaposed squamous epithelium. Vulvar pagetoid neoplasm of urothelial origin is the secondary, noncutaneous type of extramammary Paget disease, which was termed pagetoid urothelial intraepithelial neoplasia by Wilkinson and Brown [5]. Pagetoid urothelial intraepithelial neoplasia can involve the vagina, uterine cervix, and rarely the endometrium [6]. Although primary and secondary types of Paget disease have overlapping clinical presentations, histologic distinction is usually straightforward; the former is characterized by Paget cells with abundant pale cytoplasm with rare mitoses, while the latter usually displays severe disarrangement of neoplastic cells with mitotic activity often showing pleomorphism/anaplasia [6] (Table 1). However, to avoid diagnostic challenges, diagnosis of pagetoid urothelial intraepithelial neoplasia often requires detection of specific markers of urothelial differentiation. UC typically shows positivity for high-molecular-weight CK and p63 with coexpression of CK7 and CK20 in 50 to 80% of cases [7]. Both uroplakin III and thrombomodulin are highly specific to UC [7], and a newer marker, uroplakin II (clone BC21), demonstrates improved sensitivity (around 80%) and positive intensity compared with uroplakin III [8]. While a panel of these antibodies is generally sufficient for differential diagnosis, the history of prior UC provides the most important diagnostic clue for pagetoid urothelial intraepithelial neoplasia.

**Table 1:** Clinicopathologic and laboratory differences between Paget-like neoplasms and mimics of the lower genital tract

	Extramammary/vulvar Paget disease	Pagetoid urothelial carcinoma	Pagetoid colorectal carcinoma	High-risk HPV-associated neoplasm
History	Not characterized	Prior urinary tract neoplasm	Prior anorectal neoplasm	Atypical squamous/glandular cells in screening cytology
Cell morphology	Paget cells characterized by abundant pale cytoplasm and prominent nucleoli with rare mitoses	Highly stratified/disarranged or pleomorphic/anaplastic cells with mitotic activity	Colonic type cells with intracytoplasmic mucin and goblet cells	Cells indicating high-grade squamous/glandular intraepithelial lesion or their invasive forms
Immunochemical markers				
Typically positive	CK7, CAM5.2, GCDFP15, CEA, CA125, HER2/neu, androgen receptor	CKs 7 and 20 (co-expression), p63, HMWCK, uroplakins II and III, thrombomodulin	CK20, CEA, CDX2, MUC2	p40, p63, HMWCK (squamous neoplasm); CK7, CEA (glandular neoplasm)
Typically negative	CK20, ER, PgR	GCDFP15, CEA	CK7, GCDFP15	CK20, GCDFP15
p16/Ki-67 double labeling	Unknown	Usually positiveRef #9	Unknown	Positive
GATA3	PositiveRef #10	Usually positiveRef #11	NegativeRef #11	Usually negative, but occasionally weakly positiveRef #12
HPV test	Negative	Negative	Negative	High-risk genotypes detected

*HPV* human papillomavirus, *CK* cytokeratin, *ER* estrogen receptor, *PgR* progesterone receptor, *GCDFP15* gross cystic disease fluid protein 15, *CEA* carcinoembryonic antigen, *HER2* human epidermal growth factor receptor 2, *HMWCK* high-molecular-weight cytokeratin, *MUC2* mucin 2, *GATA3* GATA binding protein 3, *Ref* reference article

LBC has recently been widely applied to various samples with subsequent immunocytochemistry as a useful diagnostic tool. In the present case, the absence of p16 labeling on CINtec® *PLUS* with positivity for GATA3 led the pathologist to suspect pagetoid urothelial intraepithelial neoplasia, which was confirmed upon obtaining the clinical history. However, careful attention is needed when using these two relatively new markers because UC must be discriminated from mimics (Table 1). Piaton et al. investigated p16/Ki-67 dual labeling in urinary cytology and found p16 positivity in 93 (93.9%) of 99 high-grade urothelial cells, among which coexpression of p16/Ki-67 in the same cells was noted in 80 (79.2%) of 101 high-grade tumors [9]. It is noted that the weak p16 staining is usually considered negative. GATA3 is not useful for distinguishing pagetoid urothelial intraepithelial neoplasia from primary extramammary Paget disease; Zhao et al. recently reported that almost all 72 cases of primary vulvar Paget disease were positive for GATA3 [10]. However, GATA3 is helpful to exclude either secondary Paget disease of colorectal origin or high-risk HPV-associated neoplasm [11, 12]. Therefore, to ensure the diagnosis of pagetoid urothelial intraepithelial neoplasia, adenocarcinoma must be excluded using reliable markers such as CEA and GCDFP15 in addition to specific high-risk HPV detection in cervical cytology.

## Conclusions

In summary, we have presented an unusual case of pagetoid urothelial intraepithelial neoplasia with extension to the vagina in a woman who underwent left nephroureterectomy and subsequent radical cystectomy for metachronous UC, highlighting LBC with immunocytochemical pitfalls. Pagetoid urothelial intraepithelial neoplasia of the lower genital tract is difficult to diagnose without symptomatic lesions typical to Paget disease, such as pruritic eczema in the vulvar vestibule and/or periurethral region. Pathologists should nevertheless consider remote recurrence of UC in cervical cytology with special attention to absence of p16 labeling despite high-grade morphology as a pointer to the diagnosis of urothelial cancer. Using further biopsy and immunohistochemical confirmation of UC relapse, investigation to rule out invasive malignancies and careful follow-up throughout the patient’s lifetime is recommended.

## Abbreviations

CEA: Carcinoembryonic antigen
CK: Cytokeratin
GATA3: GATA binding protein 3
GCDFP15: Gross cystic disease fluid protein 15
HPV: Human papillomavirus
LBC: Liquid-based cytology
UC: Urothelial carcinoma
